# Genetic diversity of *Haemonchus contortus* isolated from sympatric wild blue sheep (*Pseudois nayaur*) and sheep in Helan Mountains, China

**DOI:** 10.1186/s13071-017-2377-0

**Published:** 2017-09-19

**Authors:** Dong-dong Shen, Ji-fei Wang, Dan-yu Zhang, Zhi-wei Peng, Tian-yun Yang, Zhao-ding Wang, Dwight D. Bowman, Zhi-jun Hou, Zhen-sheng Liu

**Affiliations:** 1 College of Wildlife Resources, Northeast Forestry University, Harbin, China; 2 Ningxia Helan Mountain National Nature Reserve, Yinchuan, China; 3 Inner Mongolia Helan Mountain National Nature Reserve, Alashan Left Banner, China; 4 College of Veterinary Medicine, Cornell University, Ithaca, NY USA; 5 Key Laboratory of Wildlife Conservation, China State Forestry Administration, Harbin, China

**Keywords:** Blue sheep, *Haemonchus contortus*, ITS2, *nad*4, Genetic variation, Helan Mountains

## Abstract

**Background:**

*Haemonchus contortus* is known among parasitic nematodes as one of the major veterinary pathogens of small ruminants and results in great economic losses worldwide. Human activities, such as the sympatric grazing of wild with domestic animals, may place susceptible wildlife hosts at risk of increased prevalence and infection intensity with this common small ruminant parasite. Studies on phylogenetic factors of *H. contortus* should assist in defining the amount of the impact of anthropogenic factors on the extent of sharing of agents such as this nematode between domestic animals and wildlife.

**Methods:**

*H. contortus* specimens (*n* = 57) were isolated from wild blue sheep (*Pseudois nayaur*) inhabiting Helan Mountains (HM), China and additional *H. contortus* specimens (*n* = 20) were isolated from domestic sheep that were grazed near the natural habitat of the blue sheep. Complete ITS2 (second internal transcribed spacer) sequences and partial sequences of the *nad*4 (nicotinamide dehydrogenase subunit 4 gene) gene were amplified to determine the sequence variations and population genetic diversities between these two populations. Also, 142 *nad*4 haplotype sequences of *H. contortus* from seven other geographical regions of China were retrieved from database to further examine the *H. contortus* population structure.

**Results:**

Sequence analysis revealed 10 genotypes (ITS2) and 73 haplotypes (*nad*4) among the 77 specimens, with nucleotide diversities of 0.007 and 0.021, respectively, similar to previous studies in other countries, such as Pakistan, Malaysia and Yemen. Phylogenetic analyses (BI, MP, NJ) of *nad*4 sequences showed that there were no noticeable boundaries among *H. contortus* populations from different geographical origin and population genetic analyses revealed that most of the variation (94.21%) occurred within *H. contortus* populations. All phylogenetic analyses indicated that there was little genetic differentiation but a high degree of gene flow among the *H. contortus* populations among wild blue sheep and domestic ruminants in China.

**Conclusions:**

The current work is the first genetic characterization of *H. contortus* isolated from wild blue sheep in the Helan Mountains region. The results revealed a low genetic differentiation and high degree of gene flow between the *H. contortus* populations from sympatric wild blue sheep and domestic sheep, indicating regular cross-infection between the sympatrically reared ruminants.

**Electronic supplementary material:**

The online version of this article (10.1186/s13071-017-2377-0) contains supplementary material, which is available to authorized users.

## Background

*Haemonchus contortus* is one of the major veterinary pathogens of small ruminants with a worldwide distribution [1–3]. Adult female worms of the gastrointestinal nematode are prolific and one female can produce up to 10,000 eggs per day [4]. The eggs are excreted in the feces of the host and develop on pasture to infective third-stage larvae (L3 s), which infect new hosts when ingested [5]. Clinical signs associated with infections by the hematophagous adult worms include anaemia, bottle-jaw (accumulation of serous fluid under the lower jaw), diarrhoea, even death [6]. *Haemonchus contortus* causes major economic losses in the sheep and goat industry around the world [7, 8].

Accurate identification and genetic characterization have significant practical implications for the control of this nematode [6]. Studies on genetic diversity of *H. contortus* isolated from domestic ruminants have been conducted in many geographical regions of the world, including Australia, Brazil, Europe, Malaysia, America, Pakistan and China [9–16]. Due to the many variable nuclear loci in the ITS (the ribosomal internal transcribed spacer) regions, the ITS2 sequence can be used to distinguish sympatric species of the genus *Haemonchus* [13, 15]. Also, the mitochondrial DNA (mtDNA), especially the *nad*4 (nicotinamide adenine dinucleotide dehydrogenase subunit 4) gene has been identified as a useful molecular marker for phylogenetic analysis and the most commonly used marker for studying population structure and genetic differentiation because the higher rate of substitution in this protein-coding locus makes it possible to detect differences among closely related worms [9, 15].

The Helan Mountains (HM) region is an important natural habitat of many wild ruminant species, and represents the northern distribution range of the blue sheep (*Pseudois nayaur* Hodgson, 1833) in China [17]. In the early 1980s, a thriving livestock industry with migratory herdsmen lead to habitat destruction, and the blue sheep population numbers declined sharply to a population of only about 1000 animals [18]. After relevant protective measures were taken, the number of blue sheep in HM had risen to more than 10,000 in 2004 [19]. Blue sheep is one of the most numerous and widely distributed wild ruminants in the Tibetan Plateau and is also the main prey of endangered snow leopard (*Panthera uncia* Schreber, 1775) [20]. Therefore, the population size and distribution of *P. nayaur* has always been a conservation concern in China [21].

In the case of *H. contortus*, anthropogenic factors are expected to have an impact on nematode populations in wild ruminants that are sympatric with domestic ruminants and phylogenetic studies can assist in defining the amount of influence [22]. *Pseudois nayaur* inhabiting the HM region is a species sympatric with domestic ruminants. However, no research on *Haemonchus* species of the blue sheep has been previously reported. Thus, the present study was undertaken with the following aims: (i) to assess the genetic characterization of *H. contortus* isolated from the wild blue sheep; and (ii) to explore the phylogenetic relationships of *H. contortus* isolates parasitic in the blue sheep, sheep sympatric with the blue sheep, and other domestic ruminants (sheep and goats) from different geographical regions of China.

## Methods

### Parasite material

Fifty-seven adult *Haemonchus* spp. specimens were isolated from five wild blue sheep that were culled from the population by local government agents in the areas of Qingyang Ravine (QYR) (105°54′E, 38°33′N) and Xiazi Ravine (XZR) (105°50′E, 38°29′N). Twenty adult *Haemonchus* spp. specimens were also isolated from six sheep that were randomly chosen from a mountain village near that natural habitat of blue sheep, Farm Seven Team (FST) (106°02′E, 38°31′N). All the 77 specimens were rinsed in saline, identified morphologically according to Lichtenfels et al. [23] as *Haemonchus* spp. and preserved in 70% ethanol until DNA was extracted.

### Isolation of genomic DNA, PCR amplification and sequencing

Genomic DNA from each nematode was extracted with the QIAamp DNA Mini kit (QIAamp, Hilden, Germany) following the manufacturer’s instructions. DNA samples were stored at -20 °C until use.

For species identification, a *c.*300 bp fragment of the internal transcribed spacer 2 region (ITS2) located between 5.8S and 28S regions was amplified using the conserved primers: NC1 (5′-ACG TCT GGT TCA GGG TTG TT-3′) and NC2 (5′-TTA GTT TCT TTT CCT CCG CT-3′) [24]. For genetic diversity analysis, partial (~740 bp) fragment of the mitochondrial *nad*4 was amplified by PCR using primer1-F (5′-GGA TTT GGT CAG CAA ATT GAA-3′) and primer2-R (5′-GCC TGC AAA TGA ATT AAC A-3′) [11]. PCR reactions (25 μl) were performed in 12 μl of ddH_2_O water, 2.5 μl PCR buffer, 2.5μl dNTP, 1 μl of each primer, 2 μl MgCl_2_, 3 μl DNA and 1 μl Thermo Scientific *Taq* DNA Polymerase under the following conditions for ITS2: 2 min initial denaturation at 94 °C: 30 cycles of 30 sat 94 °C,15 s at 58 °C, 30 s at 72 °C; and 7 min final extension at 72 °C; and for *nad*4: 2 min initial denaturation at 94 °C: 30 cycles of 30 s at 94 °C, 15 s at 50 °C, 1 min at 72 °C; and 7 min final extension at 72 °C.

Amplicons (5 μl) were identified on 1.0% agarose gels to verify that they represented single bands. Column-purified PCR products were sent to Comate Biosciences Co., Ltd. (Changchun, China) for sequencing. For more precision, sequencing of amplicons was done in forward and reverse directions.

### Data analysis

The raw sequences (ITS2) were aligned using E-INS-i of the program MAFFT [25]. The raw *nad*4 sequences were aligned by a codon-based alignment that was performed with the algorithm MUSCLE [26] as recommended in MEGA 5.05 [27]. Average number of nucleotide differences, haplotype diversity and nucleotide diversity were calculated with DnaSP 5.10 [28].

Phylogenetic analyses of *nad*4 sequences were performed using maximum parsimony (MP), neighbor-joining (NJ), and Bayesian inference (BI). The *nad*4 sequence for *Haemonchus placei* (GenBank: AP017687) was used as the outgroup. MP analyses were conducted using PAUP* version 4.0b10 [29]. All characters were weighted equally and unordered, and only potentially phylogenetically informative sites were retained for tree searching. Analyses used a heuristic search with 1000 random stepwise additions followed by tree bisection reconnection (TBR) branch swapping. MP bootstrap branch support values (MPBS) were calculated with 1000 pseudoreplicates with ten random-addition sequences performed in each replication. NJ analyses were conducted using MEGA 5.05, based on the Tamura-Nei model [27]. Confidence limits were assessed using bootstrap procedure with 1000 pseudoreplicates. For BI, The best-fitting nucleotide substitution model for the phylogenetic analyses was GTR + I + G which was selected by using Akaike’s information criterion, as implemented in MODELTEST3.7 [30]. Phylogenetic trees were generated using MrBayes v.3.2 [31], and parameters were set to nst = 6, rates = invgamma, four Markov chains Monte Carlo (MCMC) were run for 2 runs from random starting trees for 30 million generations, and trees were sampled every 100 generations. The 25% generations were discarded as ‘burn-in’ and the remaining samples were used to calculate Bayesian posterior probabilities (BPP).

**Fig. 1:**
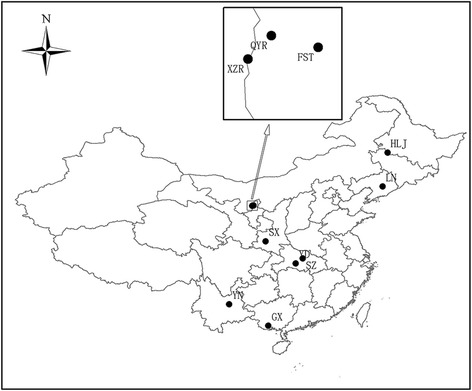
Sampling sites in this study and seven other geographical regions of China where *Haemonchus* spp. were isolated from the wild blue sheep and domestic ruminants. Qingyang Ravine and Xiazi Ravine belong to the Helan Mountains. *Abbreviations*: QYR, Qingyang Ravine; XZR, Xiazi Ravine; FST, Farm Seven Team; GX, Guangxi; HLJ, Heilongjiang; LN, Liaoning; SX, Shaanxi; SZ, Suizhou; YD, Yidu; YN, Yunnan

In order to genetically compare *H. contortus* from the wild blue sheep with that of *H. contortus* from domestic ruminants in China, another 142 *nad*4 haplotype sequences of *H. contortus* (GenBank: KC429944–KC430085) isolated from sheep and goats in seven geographical regions in China (Fig. 1), i.e. Guangxi (GX), Heilongjiang (HLJ), Liaoning (LN), Shaanxi (SX), Suizhou (SZ), Yidu (YD) and Yunnan (YN), were retrieved from the NCBI nucleotide database (http://www.ncbi.nlm.nih.gov). For all the *nad*4 sequences, multiple alignments were performed with MUSCLE as recommended in MEGA 5.05, and conserved regions were determined using the Gblocks software [32]. Phylogenetic tree was hypothesized using the above method. For BI, the best-fitting models of sequence evolution for all *nad*4 sequences was GTR + I + G. Other settings were unchanged but the MCMC was changed to 70 million generations. Phylograms were drawn using FigTree v1.4.2.

Pairwise *Fst* value, an index of genetic differentiation among populations was calculated using the program Arlequin 3.5 [33] and the statistical significance for *Fst* was established based on 1000 permutations. To directly measure and estimate gene flow between the *H. contortus* populations, *Nm* (the migration number of each generation) values were deduced according to the following formula: *Fst* = 1 / (4 *Nm* + 1) [34]. The analysis of molecular variance (AMOVA) was carried out using the program Arlequin 3.5 [33] and all *nad*4 sequences of the nine study regions (Fig. 1) in China were divided into four groups as follows: Group 1: North-east (HLJ, LN); Group 2: Central (FST, YD, SZ, SX); Group 3: HM (QYR, XZR); and Group 4: South-west (GX, YN). To further study the relationships among the haplotypes, a median joining (MJ) network was constructed using Network v4.6 [35].

## Results

### Sequence analyses

ITS2 sequences were amplified and sequenced to determine taxonomic status of the 77 *Haemonchus* spp.. After sequence editing and alignment, a complete sequence (231 bp) of the ITS2 spacer was obtained from all worms. All *Haemonchus* spp. specimens isolated from the wild blue sheep and the domestic sheep were *Haemonchus contortus*.

The analysis of the 77 ITS2 sequences revealed 10 distinct genotypes (GenBank: KY305780–KY305789). Genotype 1, 2 and 3 were found to be predominant (25.97, 22.08 and 28.57%, respectively). The multiple alignment of all 10 sequences with the reference sequence X78803 revealed eleven variable nucleotide positions, three of which (positions 21, 123 and 196) were main variation positions (Table 1). These substitutions represented six transversions (two A < − > T, two G < − > C, two T < − > G) and five transitions (T < − > C) (Table 1). The nucleotide diversities and genotype diversities of the ITS2 sequences from domestic sheep and wild blue sheep were 0.004 and 0.008, and 0.719 and 0.787, respectively (Table 2).

**Table 1:** Nucleotide details and distribution of 10 ITS2 genotypes from 77 *H. contortus* worms from blue sheep and sheep in this study. Points represent similar positions

Genotype	Position											Number of parasites		
	18	19	21	22	45	59	63	78	79	123	196	Blue sheep	Sheep	Total
X78803	T	G	C	T	T	T	C	T	T	C	A	–	–	–
GT1	•	•	G	•	•	•	•	•	•	T	T	12	8	20
GT2	•	•	•	•	•	•	•	•	•	T	T	12	5	17
GT3	•	•	G	C	•	•	•	•	•	T	T	17	5	22
GT4	•	•	G	•	•	•	•	•	•	T	•	2	1	3
GT5	•	•	G	C	•	•	•	•	•	T	•	3	0	3
GT6	A	•	G	C	•	•	T	C	•	•	•	4	0	4
GT7	A	•	G	C	•	G	T	C	•	•	•	1	1	2
GT8	•	•	•	•	G	•	•	•	C	T	T	2	0	2
GT9	•	C	•	•	•	•	•	•	•	T	T	3	0	3
GT10	•	•	•	•	G	•	•	•	•	T	T	1	0	1
Total												57	20	77

**Table 2:** Details of nucleotide, haplotype diversity and neutrality for *H. contortus* population in sheep and blue sheep

Host	ITS2			*nad*4					
	*N*	Gd	π	Ps	Pi	Si	h	Hd	π
Sheep	20	0.719	0.004	61	36	25	18	0.995	0.020
Blue sheep	57	0.787	0.008	114	60	54	55	0.999	0.021
Total	77	0.767	0.007	127	66	67	73	0.999	0.021

*Abbreviations*: *N* *H. contortus* population size, *GD* genotype diversity, π nucleotide diversity, *Ps* polymorphic sites, *Pi* parsimony informative sites, *Si* singleton sites, *h* number of haplotypes, *Hd* haplotype diversity

The *nad*4 gene of all 77 *H. contortus* was successfully amplified. After sequences editing and alignment, a 744 bp gene fragment was obtained that was consistent with base position 13,113 to 13,858 of the complete mitochondrial genome of *H. contortus* (GenBank: NC_010383). A high degree of *nad*4 gene diversity was observed within the two *H. contortus* populations from domestic sheep and wild blue sheep. Nucleotide diversity of *H. contortus* populations from sheep and wild blue sheep was 0.020 and 0.021, respectively. A total of 127 polymorphic sites and 66 parsimony informative sites were observed. Analysis of 77 individual nematodes for the *nad*4 gene revealed 73 unique haplotypes. Herein 55 haplotypes (Table 2) were detected from the wild blue sheep *H. contortus* population. Those haplotypes showed one shared haplotype between the sheep and the wild blue sheep *H. contortus* populations; additionally, the haplotype diversity within the two populations was very high (0.995 and 0.999, respectively). All of the haplotype sequences were submitted to GenBank under the accession numbers (KY305790–KY305862).

### Phylogenetic analysis of the *nad*4 gene

**Fig. 2:**
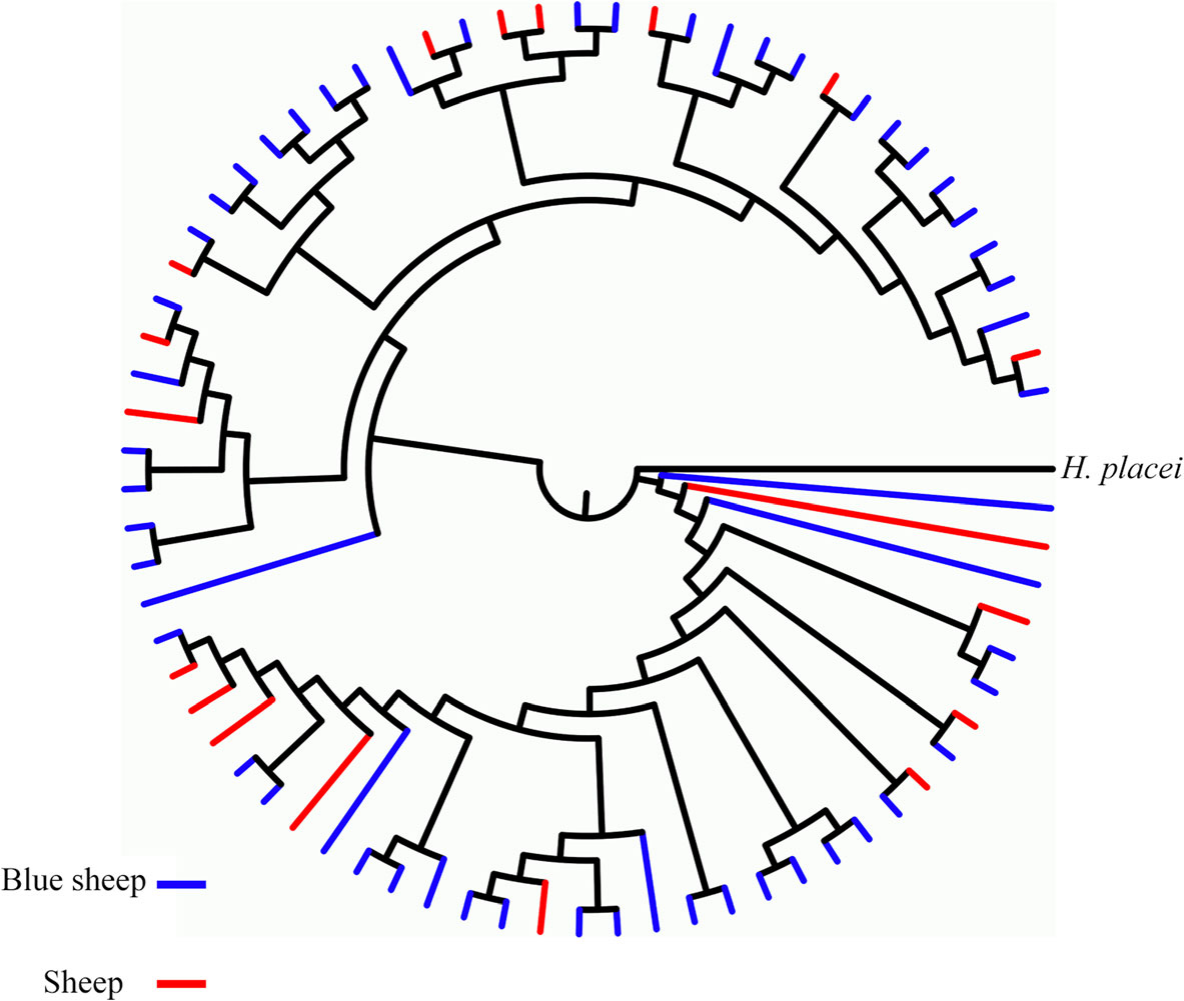
Phylogenetic tree resulting from Bayesian analysis (30 million generations) constructed with 55 *nad*4 haplotype sequences from wild blue sheep in Helan Mountains and 18 haplotypes from the sheep in Farm Seven Team. The likelihood values of the 50% majority consensus tree was lnL = -3431.443. The average standard deviation of split frequencies was 0.004003 and all ESS values > 200. The different coloured branches represent haplotypes from the different sampling locations/hosts. Posterior probabilities were too low and not displayed in the tree

A phylogenetic tree (Fig. 2) resulting from Bayesian analysis (30 million generations) was constructed with 55 *nad*4 haplotypes sequences from wild blue sheep and 18 haplotypes from domestic sheep. The dendrogram revealed that the 18 haplotypes of *H. contortus* from sheep randomly dispersed among haplotypes of *H. contortus* from wild blue sheep, with poorly supported nodes. There were no clear boundaries among nematode individuals from the two hosts in the tree. The MP and NJ trees (data not shown) had almost similar topologies. Additional file 1: Figure S1 shows the location of the *nad*4 sequences representing *H. contortus* specimens from every blue sheep located within the phylogenetic tree.

**Fig. 3:**
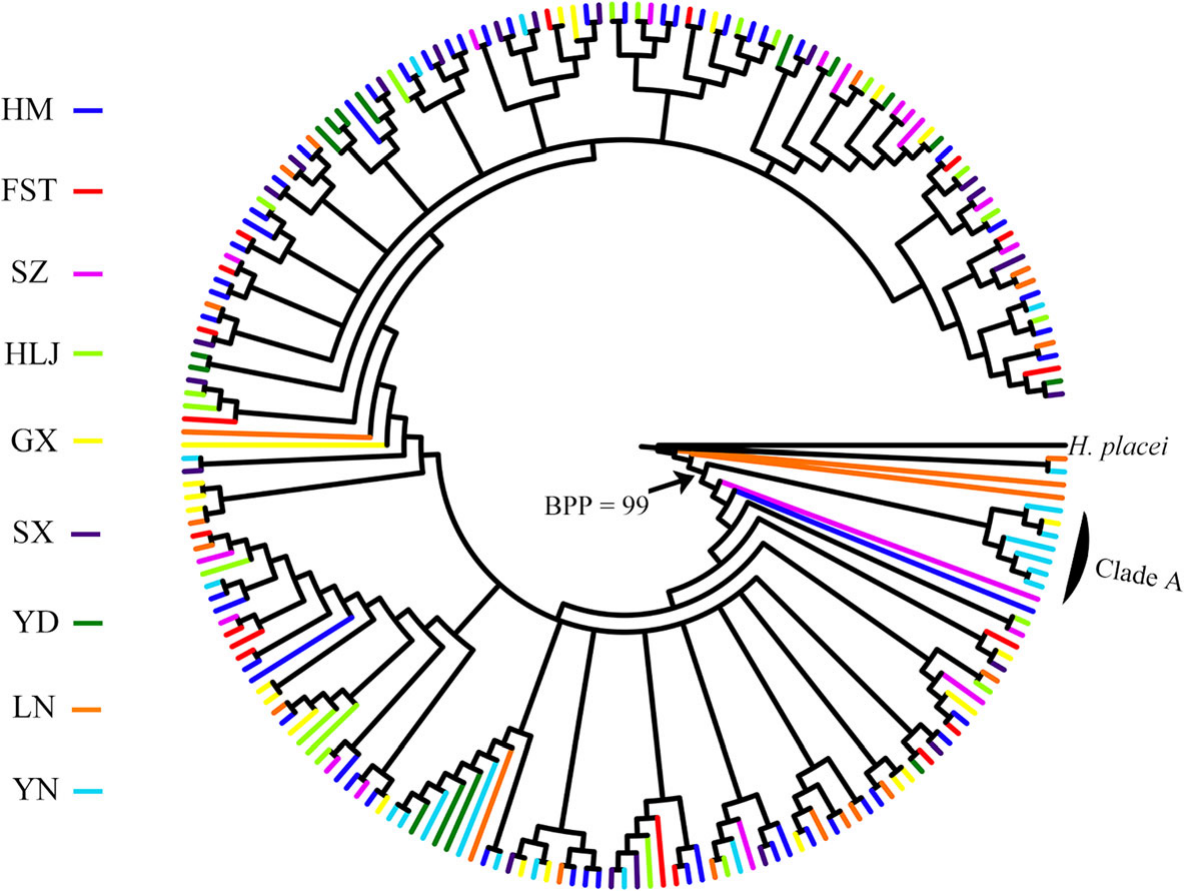
Phylogenetic tree resulting from Bayesian analysis (70 million generations) of 215 *nad*4 sequences of *H. contortus* (55 from HM, 18 from FST, 21 from SZ, 15 from YD, 21 from GX, 20 from YN, 23 from SX, 22 from LN, 20 from HLJ). The likelihood values of the 50% majority consensus tree was lnL = -3971.767. The average standard deviation of split frequencies was 0.007252 and all ESS values >200. The different coloured branches represent *nad*4 sequences from the different populations/sampling locations. Posterior probabilities were too low and not displayed in the tree. *Abbreviations*: FST, Farm Seven Team; GX, Guangxi; HLJ, Heilongjiang; HM, Helan Mountains; LN, Liaoning; SX, Shaanxi; SZ, Suizhou; YD, Yidu; YN, Yunnan

To further determine the genetic relationships of *H. contortus* from the wild blue sheep and domestic ruminants, a phylogenetic tree of 215 *nad*4 sequences of *H. contortus* (55 individuals from blue sheep, 18 individuals from sheep sympatric with the blue sheep and 142 individuals isolated from sheep and goats in the seven different geographical regions of China) was constructed by the three methods. The topology of the BI tree showed (Fig. 3) that six individuals from YN population and one GX individual formed a distinct clade (Clade A) with high prior probability (BPP = 0.99), which was consistent with study of Yin et al. [16]. Sequences representing the *H. contortus* individuals isolated from the wild blue sheep were randomly dispersed in the branches of the tree with weak support. Corresponding with the BI inference, the MP and NJ methods also showed the same results (not shown) that no noticeable boundaries were present in the trees among the haplotype sequences. Additional file 2: Figure S2 also shows the location of the *nad*4 sequences representing *H. contortus* specimens from every blue sheep on the phylogenetic tree.

**Fig. 4:**
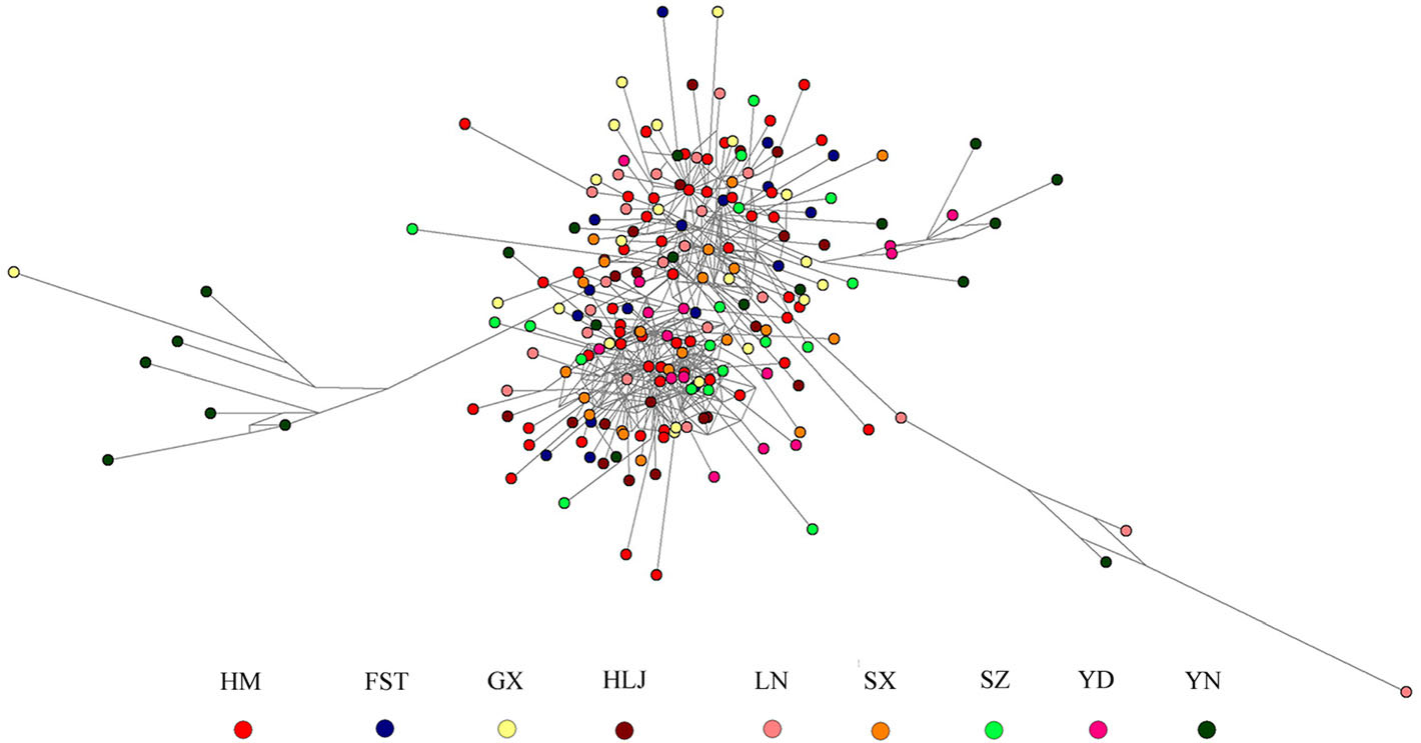
Median joining network of *nad*4 haplotypes derived from *H. contortus* isolated from wild blue sheep, sheep sympatric with the blue sheep and seven geographical regions of China. The different coloured dots represent haplotypes from the different populations/sampling locations. *Abbreviations*: FST, Farm Seven Team; GX, Guangxi; HLJ, Heilongjiang; HM, Helan Mountains; LN, Liaoning; SX, Shaanxi; SZ, Suizhou; YD, Yidu; YN, Yunnan

The median joining (MJ) network of *nad*4 haplotypes was constructed to discern the relationships of *H. contortus* from wild blue sheep and domestic ruminants. From the network profile (Fig. 4), there was no obvious grouping among *H. contortus* individuals from different geographical regions, except for several YN individuals. A close genetic relationship of all *H. contortus* individuals from wild blue sheep and domestic ruminants was revealed in the network profile.

### Population genetic structure

Furthermore, pairwise *F*st values and *Nm* values were also calculated among the *H. contortus* populations isolated from the HM and other geographical regions in China. *F*st value between the *H. contortus* populations isolated from wild blue sheep and sheep (in FST) was 0 and the *Nm* value was infinite, which indicated relative homogeneity. The wild blue sheep *H. contortus* population and domestic ruminants *H. contortus* populations from the seven geographical regions of China showed low level in *F*st values, ranging from 0 to 0.23262. Similarly, low *Fst* values were also shown between the sheep (in FST) *H. contortus* population and the seven geographical populations from domestic ruminants (Table 3). All *Nm* values among these *H. contortus* populations were greater than 1, except for the *Nm* value of 0.82471 between HM and YN populations (Table 4).

**Table 3:** Pairwise *Fst* values of partial *nad*4 gene between *H. contortus* populations from the nine regions in China

	FST	HLJ	LN	SX	SZ	YD	GX	YN
FST	–	0	0.01147	0.03446	0	**0.06616**	0	**0.16233**
HM	0	0	**0.03090**	0.00740	0.01234	**0.06021**	0.03100	**0.23262**

Bold indicates significant *Fst* values (*P* < 0.05)

*Abbreviations*: *FST* Farm Seven Team, *GX* Guangxi, *HLJ* Heilongjiang, *HM* Helan Mountains, *LN* Liaoning, *SX* Shaanxi, *SZ* Suizhou, *YD* Yidu, *YN* Yunnan

**Table 4:** Migration number of each generation (*Nm*) values among *H. contortus* populations deduced based on *Fst* = 1 / (4 *Nm* + 1)

	FST	HLJ	LN	SX	SZ	YD	GX	YN
FST	–	Inf	21.54599	7.00479	Inf	3.52872	Inf	1.29007
HM	Inf	Inf	7.840615	33.53378	20.00932	3.90214	7.81452	0.82471

*Abbreviations*: *Inf* the *Nm* values are infinite, *FST* Farm Seven Team, *GX* Guangxi, *HLJ* Heilongjiang, *HM* Helan Mountains, *LN* Liaoning, *SX* Shaanxi, *SZ* Suizhou, *YD* Yidu, *YN* Yunnan

The analysis of molecular variance (AMOVA) was calculated to estimate gene flow among *H. contortus* populations in China. With the hierarchical levels, the results showed a significant variation (94.21%) within populations and the lowest variation (0.44%) among groups (Table 5). The average *F*st value within populations was low (0.05788), which was greatest compared with the *Fct* value (0.00437) among groups and the *Fsc* value (0.05374) within groups.

**Table 5:** AMOVA results for percentage of variation and *F-*statistics of partial *nad*4 gene

Source of variation	Percentage of variation	*F*-statistics	*P*-value
Among groups	0.43705	Fct = 0.00437	0.19453
Among populations within groups	5.35100	Fsc = 0.05374	< 0.00001
Within populations	94.21195	Fst = 0.05788	< 0.00001

## Discussion

To our knowledge, the present study is the first to characterize genetically *Haemonchus* spp. specimens isolated from wild blue sheep inhabiting Helan Mountains, China. Complete ITS2 sequences of the 77 worms confirmed their identification as *H. contortus*. As for ITS2 sequences of individual worms from the wild blue sheep and domestic sheep, the sequence variation was 4.8 and 3.5%, respectively. These values are higher than that (2.6%) detected in *H. contortus* populations isolated from other locations, such as Sweden/Kenya [36], Germany [37] and the seven geographical regions of China [16], but lower than that (5.2%) detected in other study sites including the UK, New Zealand, Switzerland and France [10]. In the present study, ten different ITS2 genotypes and 11 polymorphic loci were observed among the *H. contortus* specimens from the wild blue sheep and sheep. Variable loci 123 and 196 of ITS2 sequences were commonly observed in other studies [14, 15]. Variations at loci 18, 21 and 22 were reported in the seven geographical regions of China [16] and variations at positions 59, 63 and 78 were observed in isolates from Pakistan [38]. As for the variable loci 19, 45 and 79 in the ITS2 region of *H. contortus* isolated from the wild blue sheep, those polymorphic loci have not been previously reported, to the best of our knowledge. For *nad*4 sequences of individual worms from the wild blue sheep and domestic sheep, the nucleotide diversity was 2.1 and 2.0% respectively; these values are in accordance with earlier published data on *H. contortus* isolated from other locations, such as the seven geographical regions of China (1.8–3.7%) [16] and Yemen (2.1–3.6%) [15]. Similarly, our study also revealed an equally high degree in haplotype diversity of the two *H. contortus* populations (Table 2) which were in accordance with previous studies on strongyloid nematodes ranging between 0.833–0.999 [39–41]. The high magnitude of diversity, as evidenced by the 73 haplotypes identified from 77 *H. contortus* individuals in our study, is typical for trichostrongyloid nematodes [42]. Yin et al. [16] found 142 haplotypes out of 152 *H. contortus* sequenced for *nad*4 sequences. In a similar study, Gharamah et al. [15] found 113 haplotypes among 120 *H. contortus* individuals.

Population genetic structure of the *nad*4 gene at different hierarchical levels showed that the majority (94.21%) of genetic variance was distributed within populations of *H. contortus* from different Chinese regions (Fig. 1) while only 0.43705% could be attributed to differences among groups. The phylogenetic tree (Fig. 2) based on 73 *nad*4 haplotypes sequences from wild blue sheep and domestic sheep revealed no obvious grouping between the two *H. contortus* populations. The phylogenetic analysis supports the lack of host specificity for *H. contortus* as reported in many studies [13, 15]. Further phylogenetic analysis (Fig. 3) based on 215 *nad*4 sequences indicated that there was no clear grouping of the *H. contortus* populations according to geographical origin (except for several specimens from YN), and the MJ network of *nad*4 haplotypes also showed the same phenomenon. This phenomenon was also supported by the low pairwise *Fst* values which were distributed among the *H. contortus* populations of HM, FST and the other regions in China. Besides, *Nm* value between the wild blue sheep and sheep in FST *H. contortus* populations was infinite and almost all *Nm* values were greater than one (Table 5). All the above results suggest that low genetic differentiation and high gene flow without clear geographical barriers appeared not only between the two newly obtained populations but also among all *H. contortus* populations.

In 2013, Yin et al. [16] had confirmed that there was no clear phylogeographic structuring among *H. contortus* populations isolated from sheep and goats in the seven geographical regions (LN, HLJ, SZ,YD, SX, GX, YN) in China, and that high gene flow was occurring across those *H. contortus* populations in concordance with host movement. Consequently, we assumed that the high level of gene flow appeared between the *H. contortus* population isolated from FST and the seven regional populations because of random movement of hosts. Besides host movement, for the parasitic nematodes, the gene flow is also determined by the life histories of both the parasite and its host, effective population sizes and multiple host species being reared together in common grazing pastures [38]. A study on *Haemonchus* species by Hoberg et al. [22] has also paid attention to wild animals sympatric with livestock and assessed implications of the anthropogenic factors that could be more significant in ecosystems affected by the human movement of livestock and random ranging of wild species [12, 43]. The Helan Mountains and the surrounding areas are the main pastoral areas in Ningxia Province, China, and the number of sheep grazed on natural grassland reached to 0.38 million by the end of 2003 [44]. Every year, approximately 100,000 sheep were observed on the mountains in June and August [45]. Considering the high fecundity and life history of *H. contortus* [4, 5], high pasture contamination might occur in the common grazing pastures where sympatric wild blue sheep and domestic ruminants feed. Taken together, domestic ruminants were grazed in the natural habitat of the wild blue sheep which may explain the phenomenon that no obvious genetic differentiation and high level of gene flow appeared between *H. contortus* populations isolated from sympatric wild blue sheep and sheep. Meanwhile, the results of genetic structure and gene flow reported here revealed that cross-infection appeared between sympatric wild blue sheep and domestic sheep. As for high gene flow between *H. contortus* populations from the wild blue sheep and domestic ruminants in the seven geographical regions of China, we hypothesize that random movement of the domestic ruminants sympatric with the wild blue sheep act as the medium for genetic exchange. It is also anticipated that the wild blue sheep as reservoir hosts of *H. contortus*, threatens sympatric wild ruminants such as *Procapra gutturosa*, *Naemorhedus goral* and *Ovis ammon* [43]. Thus, further investigations are warranted to take into account in the design of an effective control strategy.

## Conclusions

To our knowledge, the present study is the first to characterize genetically *H. contortus* specimens isolated from wild blue sheep inhabiting Helan Mountains in China. The results indicated low genetic differentiation and a high degree of gene flow occurring among *H. contortus* populations in the wild blue sheep, domestic sheep sympatric with the wild blue sheep, and among seven different regional *H. contortus* populations within China. This revealed that cross-infection occurs between sympatric wild blue sheep and domestic sheep, and that the domestic ruminants sympatric with the wild blue sheep act as a medium for high gene flow between the wild blue sheep *H. contortus* populations and other geographical populations in China. These findings may provide a reference basis for protecting wild ruminants in the Helan Mountains region against *H. contortus*.

## Additional files

Additional file 1: **Figure S1.** Phylogenetic tree resulting from Bayesian analysis constructed with 55 *nad*4 haplotype sequences from the five wild blue sheep (blue sheep A, blue sheep B, sheep C, blue sheep D and sheep E) in Helan Mountains and 18 haplotypes from the sheep in Farm Seven Team (shown in Fig. 2) showing the location of the *nad*4 sequences representing *H. contortus* specimens from every blue sheep. The different coloured branches represent haplotypes from the different wild blue sheep. *Abbreviations*: A, blue sheep A; B, blue sheep B; C, blue sheep C; D, blue sheep D; E, blue sheep E (TIFF 328 kb)

Additional file 2: **Figure S2.** Phylogenetic tree resulting from Bayesian analysis constructed with 55 *nad*4 haplotype sequences from the five wild blue sheep (blue sheep A, blue sheep B, sheep C, blue sheep D and sheep E) in Helan Mountains and 160 haplotypes from domestic ruminants (shown in Fig. 3) showing the location of the *nad*4 sequences representing *H. contortus* specimens from every blue sheep. The different coloured branches represent haplotypes from the different wild blue sheep. *Abbreviations*: A, wild blue sheep A; B, wild blue sheep B; C, wild blue sheep C; D, wild blue sheep D; E, wild blue sheep E (TIFF 490 kb)

## Abbreviations

AMOVA: Analysis of molecular variance
BI: Bayesian inference
BPP: Bayesian posterior probability
FST: Farm Seven Team
GX: Guangxi
HLJ: Heilongjiang
HM: Helan Mountains
ITS2: Second internal transcribed spacer of ribosomal DNA
LN: Liaoning
MJ: Median-joining
MP: Maximum parsimony
MPBS: MP bootstrap branch support values
*nad*4: Mitochondrial nicotinamide adenine dinucleotide dehydrogenase subunit 4 gene
NJ: Neighbour-joining
*Nm*: Migration number of each generation
QYR: Qingyang Ravine
SX: Shaanxi
SZ: Suizhou
TBR: Tree bisection reconnection
XZR: Xiazi Ravine
YD: Yidu
YN: Yunnan

## References

[1] Gibbs HC, Herd RP (1986). Nematodiasis in cattle. Importance, species involved, immunity, and resistance. Vet Clin North Am Food Anim Pract.

[2] Anderson TJ, Blouin MS, Beech RN (1998). Population biology of parasitic nematodes: applications of genetic markers. Adv Parasitol.

[3] O’Connor LJ, Walkden-Brown SW, Kahn LP (2006). Ecology of the free-living stages of major trichostrongylid parasites of sheep. Vet Parasitol.

[4] Prichard R (2001). Genetic variability following selection of *Haemonchus contortus* with anthelmintics. Trends Parasitol.

[5] Nikolaou S, Gasser RB (2006). Prospects for exploring molecular developmental processes in *Haemonchus contortus*. Int J Parasitol.

[6] Gasser RB, Bott NJ, Chilton NB, Hunt P, Beveridge I (2008). Toward practical, DNA-based diagnostic methods for parasitic nematodes of livestock - bionomic and biotechnological implications. Biotechnol Adv.

[7] Peter JW, Chandrawathani P (2005). *Haemonchus contortus*. parasite problem No. 1 from Tropics - Polar Circle. Problems and prospects for control based on epidemiology. Trop Biomed.

[8] Garretson PD, Hammond EE, Craig TM, Holman PJ (2009). Anthelmintic resistant *Haemonchus contortus* in a giraffe (*Giraffa camelopardalis*) in Florida. J Zoo Wildl Med.

[9] Blouin MS, Yowell CA, Courtney CH, Dame JB (1995). Host movement and the genetic structure of populations of parasitic nematodes. Genetics.

[10] Gasser RB, Zhu X, Chilton NB, Newton LA, Nedergaard T, Guldberg P (1998). Analysis of sequence homogenisation in rDNA arrays of *Haemonchus contortus* by denaturing gradient gel electrophoresis. Electrophoresis.

[11] Troell K, Engström A, Morrison DA, Mattsson JG, Höglund J (2006). Global patterns reveal strong population structure in *Haemonchus contortus*, a nematode parasite of domesticated ruminants. Int J Parasitol.

[12] Hunt PW, Knox MR, Le JL, Mcnally J, Anderson LJ (2008). Genetic and phenotypic differences between isolates of *Haemonchus contortus* in Australia. Int J Parasitol.

[13] Cerutti MC, Citterio CV, Bazzocchi C, Epis S, D'Amelio S, Ferrari N (2010). Genetic variability of *Haemonchus contortus* (Nematoda: Trichostrongyloidea) in alpine ruminant host species. J Helminthol.

[14] Brasil BS, Nunes RL, Bastianetto E, Drummond MG, Carvalho DC, Leite RC (2012). Genetic diversity patterns of *Haemonchus placei* and *Haemonchus contortus* populations isolated from domestic ruminants in Brazil. Int J Parasitol.

[15] Gharamah AA, Azizah MN, Rahman WA (2012). Genetic variation of *Haemonchus contortus* (Trichostrongylidae) in sheep and goats from Malaysia and Yemen. Vet Parasitol.

[16] Yin F, Gasser RB, Li F, Bao M, Huang W, Zou F (2013). Genetic variability within and among *Haemonchus contortus* isolates from goats and sheep in China. Parasit Vectors.

[17] Liu ZS, Wang XM, Li ZG, Zhai H, Hu TH (2007). Distribution and abundance of blue sheep in Helan Mountains, China. Chinese J Zool.

[18] Fox JL, Mathiesen P, Yangzom D, Nss MW (2004). Modern wildlife conservation initiatives and the pastoralist/hunter nomads of northwestern Tibet. Rangifer.

[19] Liu ZS, Cao LR, Wang XM (2004). The management and protection of Ningxia Helan mountain blue sheep population. Chinese J Wildl.

[20] Schaller GB (2000). Wildlife of the Tibetan Steppes.

[21] Schaller GB. Mountain monarchs. Wild sheep and goats of the Himalaya. Q Rev Biol. 1978:43–53.

[22] Hoberg EP, Lichtenfels JR, Gibbons L (2004). Phylogeny for species of *Haemonchus* (Nematoda: Trichostrongyloidea): considerations of their evolutionary history and global biogeography among Camelidae and Pecora (Artiodactyla). J Parasitol.

[23] Lichtenfels JR, Pilitt PA, Hoberg EP (1994). New morphological characters for identifying individual specimens of *Haemonchus* spp. (Nematoda: Trichostrongyloidea) and a key to species in ruminants of North America. J Parasitol.

[24] Stevenson LA, Chilton NB, Gasser RB (1995). Differentiation of *Haemonchus placei* from *H. contortus* (Nematoda: Trichostrongylidae) by the ribosomal DNA second internal transcribed spacer. Int J Parasitol.

[25] Katoh K, Standley D (2013). MAFFT multiple sequence alignment software version improvements in performance and usability. Mol Biol Evol.

[26] Edgar RC (2004). MUSCLE: multiple sequence alignment with high accuracy and high throughput. Nucleic Acids Res.

[27] Tamura K, Peterson D, Peterson N, Stecher G, Nei M, Kumar S (2011). MEGA5: Molecular Evolutionary Genetics Analysis using maximum likelihood, evolutionary distance, and maximum parsimony methods. Mol Biol Evol.

[28] Librado P, Rozas J (2009). DnaSP v5: a software for comprehensive analysis of DNA polymorphism data. Bioinformatics.

[29] Swofford D. PAUP: Phylogenetic Analysis Using Parsimony (*and Other Methods).Version 4.0b10. Sunderland: Sinauer Associates; 2002.

[30] Posada D, Crandall KA (1998). MODELTEST: testing the model of DNA substitution. Bioinformatics.

[31] Ronquist F, Huelsenbeck JP (2003). MrBayes 3: Bayesian phylogenetic inference under mixed models. Bioinformatics.

[32] Talavera G, Castresana J (2007). Improvement of phylogenies after removing divergent and ambiguously aligned blocks from protein sequence alignments. Syst Biol.

[33] Excoffier L, Lischer HE (2010). Arlequin suite ver 3.5: a new series of programs to perform population genetics analyses under Linux and Windows. Mol Ecol Resour.

[34] Beebee TJC, Rowe G (2008). An Introduction to Molecular Ecology.

[35] Bandelt HJ, Forster P, Röhl A (1999). Median-joining networks for inferring intraspecific phylogenies. Mol Biol Evol.

[36] Troell K, Mattsson JG, Alderborn A, Höglund J (2003). Pyrosequencing analysis identifies discrete populations of *Haemonchus contortus* from small ruminants. Int J Parasitol.

[37] Heise M, Epe C, Schnieder T (1999). Differences in the second internal transcribed spacer (ITS-2) of eight species of gastrointestinal nematodes of ruminants. J Parasitol.

[38] Hussain T, Periasamy K, Nadeem A, Babar ME, Pichler R, Diallo A (2014). Sympatric species distribution, genetic diversity and population structure of *Haemonchus* isolates from domestic ruminants in Pakistan. Vet Parasitol.

[39] Blouin MS, Yowell CA, Courtney CH, Dame JB (1998). Substitution bias, rapid saturation, and the use of mtDNA for nematode systematics. Mol Biol Evol.

[40] Hu M, Chilton NB, Abs El-Osta YG, Gasser RB (2003). Comparative analysis of mitochondrial genome data for *Necator americanus* from two endemic regions reveals substantial genetic variation. Int J Parasitol.

[41] van der Veer M, Kanobana K, Ploeger HW, de Vries E (2003). Cytochrome oxidase *c* subunit 1 polymorphisms show significant differences in distribution between a laboratory maintained population and a field isolate of *Cooperia oncophora*. Vet Parasitol.

[42] Archie EA, Ezenwa VO (2011). Population genetic structure and history of a generalist parasite infecting multiple sympatric host species. Int J Parasitol.

[43] Liu X, Zhai H, Liu HJ, Zhou QG (2004). Analysis and surveying of the species of vertebrate in Helan Mountain Natural Reserve in Ningxia. J Agric Sci.

[44] Guo LH, He TH, Qiu KY (2010). Grassland ecology and livestock production in Ningxia Province. Agric Eng Technol.

[45] Wang XM, Liu ZX, Li M (1998). The blue sheep population ecology and its conservation in Helan Mountains, China. Chinese Biodivers.

