# Supplementary figures and images for: Genetic diversity of Haemonchus contortus isolated from sympatric wild blue sheep (Pseudois nayaur) and sheep in Helan Mountains, China
Source: Parasit Vectors. 2017 Sep 19;10:437. doi: 10.1186/s13071-017-2377-0 (PMC5606089; doi:10.1186/s13071-017-2377-0)

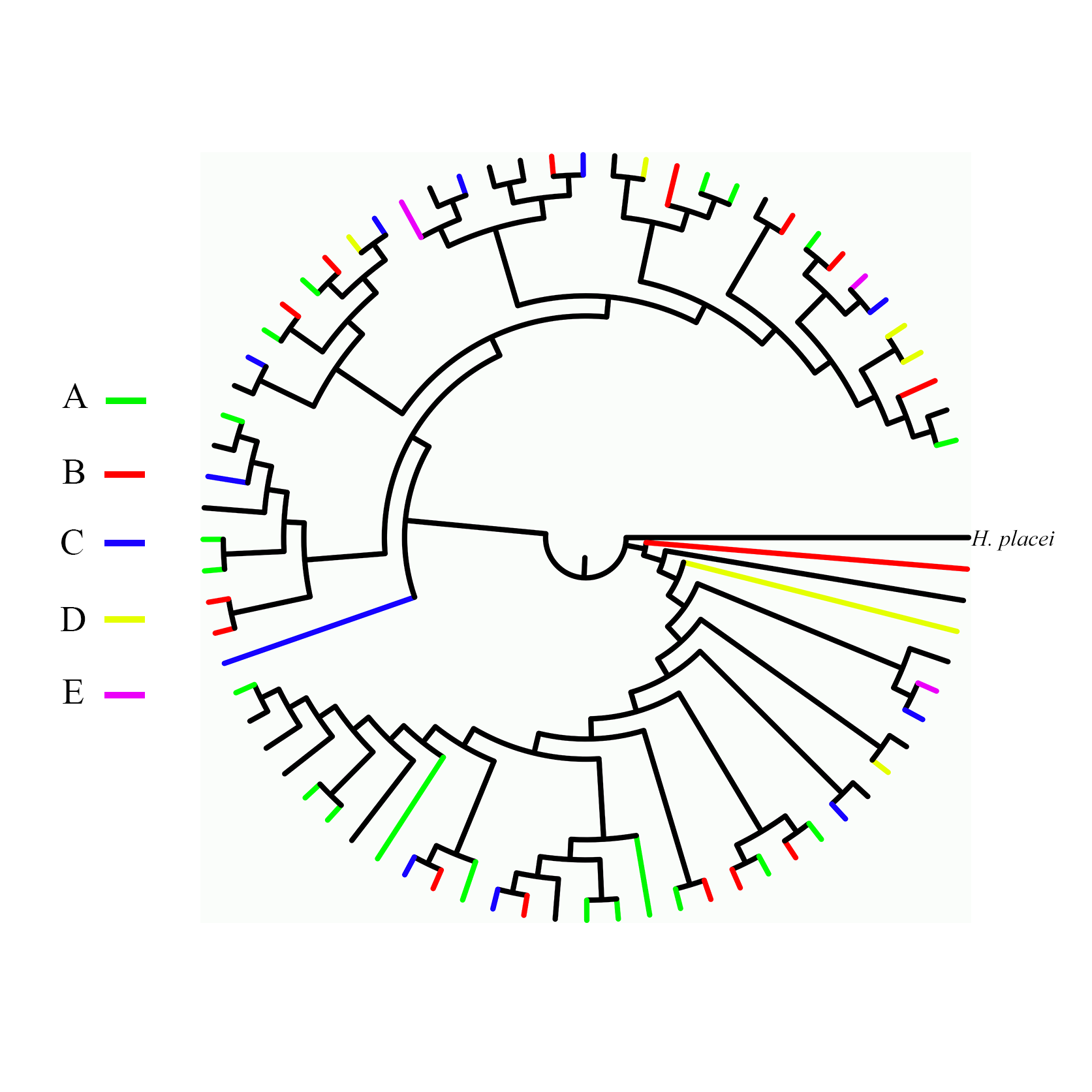

Supplement: Supplementary file 1 — Figure S1. Phylogenetic tree resulting from Bayesian analysis constructed with 55 nad4 haplotype sequences from the five wild blue sheep (blue sheep A, blue sheep B, sheep C, blue sheep D and sheep E) in Helan Mountains and 18 haplotypes from the sheep in Farm Seven Team (shown in Fig. 2) showing the location of the nad4 sequences representing H. contortus specimens from every blue sheep. The different coloured branches represent haplotypes from the different wild blue sheep. Abbreviations: A, blue sheep A; B, blue sheep B; C, blue sheep C; D, blue sheep D; E, blue sheep E (TIFF 328 kb) [file 13071_2017_2377_MOESM1_ESM.tif]

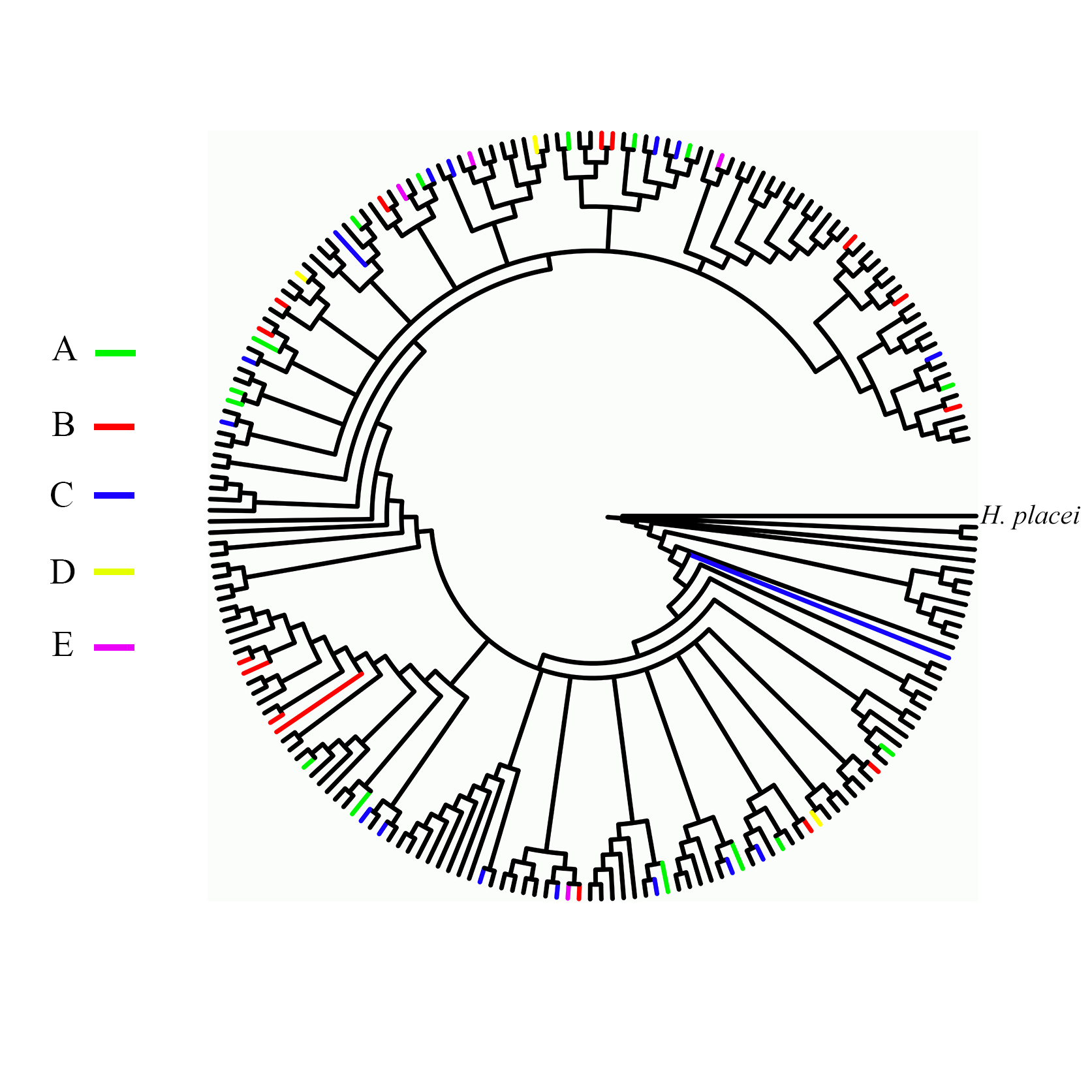

Supplement: Supplementary file 2 — Figure S2. Phylogenetic tree resulting from Bayesian analysis constructed with 55 nad4 haplotype sequences from the five wild blue sheep (blue sheep A, blue sheep B, sheep C, blue sheep D and sheep E) in Helan Mountains and 160 haplotypes from domestic ruminants (shown in Fig. 3) showing the location of the nad4 sequences representing H. contortus specimens from every blue sheep. The different coloured branches represent haplotypes from the different wild blue sheep. Abbreviations: A, wild blue sheep A; B, wild blue sheep B; C, wild blue sheep C; D, wild blue sheep D; E, wild blue sheep E (TIFF 490 kb) [file 13071_2017_2377_MOESM2_ESM.tif]
